# Relationships of* SLC2A4*,* RBP4*,* PCK1,* and* PI3K* Gene Polymorphisms with Gestational Diabetes Mellitus in a Chinese Population

**DOI:** 10.1155/2019/7398063

**Published:** 2019-01-20

**Authors:** Shimin Hu, Shujuan Ma, Xun Li, Zhengwen Tian, Huiling Liang, Junxia Yan, Mengshi Chen, Hongzhuan Tan

**Affiliations:** ^1^School of Public Health, Central South University, 90 Xiangya Road, Changsha, Hunan, China; ^2^Department of Obstetrics and Gynecology, Xiangya Hospital of Central South University, 87 Xiangya Road, Changsha, Hunan, China

## Abstract

**Background:**

Solute carrier family 2 member 4- (SLC2A4-) retinol binding protein-4- (RBP4-) phosphoenolpyruvate carboxykinase 1 (PCK1)/phosphoinositide 3-kinase (PI3K) is an adipocyte derived “signalling pathway” that may contribute to the pathogenesis of type 2 diabetes mellitus (T2DM). We explored whether single nucleotide polymorphisms (SNPs) of these “signalling pathway” genes are associated with gestational diabetes mellitus (GDM).

**Methods:**

Case-control studies were conducted to compare GDM and control groups. A total of 334 cases and 367 controls were recruited. Seventeen candidate SNPs of the pathway were selected. Chi-square tests, logistic regression, and linear regression were used to estimate the relationships of SNPs with GDM risk and oral glucose tolerance test (OGTT), fasting insulin, and homeostasis model assessment of insulin resistance (HOMA-IR) levels. Model-based multifactor dimensionality reduction was used to estimate the adjusted interactions between genes. Regression and interaction analyses were adjusted by maternal age, prepregnancy BMI, and weekly BMI growth. The Bonferroni correction was applied for multiple comparisons.

**Results:**

* RBP4* rs7091052 was significantly associated with GDM risk.* SLC2A4* rs5435,* RBP4* rs7091052,* PCK1* rs1042531 and rs2236745, and* PIK3R1 *(coding gene of the PI3K P85 subunit) rs34309 were associated with OGTT, fasting insulin, and HOMA-IR levels in the linear regression analysis. The gene-gene interaction analysis showed that, compared with pregnant women with other genotype combinations, women with* SLC2A4 *rs5435 (CC/CT),* RBP4 *rs7091052 (CC),* PCK1* rs1042531 (TT/TG) and rs2236745 (TT), and* PIK3R1 *rs34309 (AA) had lower GDM risk.

**Conclusion:**

* SLC2A4*,* RBP4*,* PCK1, *and* PIK3R1* genes may be involved in the pathogenesis of GDM.

## 1. Introduction

Gestational diabetes mellitus (GDM) is defined as varying degrees of glucose intolerance that is first detected during pregnancy [1]. The prevalence of GDM has increased in recent decades, ranging from 1.7 to 11.6% among various populations [2]. During pregnancy, because the placenta secretes a series of hormones with an insulin antagonistic function, such as progesterone, prolactin, oestrogen, and cortisol, pregnant women appear physiologically insulin resistant and secrete more insulin to maintain normal blood glucose levels. When insulin resistance reaches higher levels, the insulin compensatory secretion becomes insufficient, blood glucose rises, and GDM occurs. Severe insulin resistance is the core of GDM pathophysiology[3]. To date, it has been widely accepted that the molecular mechanism of insulin resistance is mainly associated with post-insulin-receptor signal transduction defects. The “substrate" proteins that are phosphorylated by insulin receptors include a protein known as insulin receptor substrate 1 (IRS-1). IRS-1 binding and phosphorylation eventually lead to increased levels of high affinity glucose transfer protein-4 (also known as solute carrier family 2 member 4, SLC2A4) molecules on the outer membrane of insulin-responsive tissues and, therefore, increased glucose uptake from blood into these tissues. Disturbance of any of the abovementioned processes can affect the signal transduction of insulin, leading to insulin resistance[4].

In obesity and type 2 diabetes mellitus (T2DM), the expression of* SLC2A4* is selectively decreased in adipocytes. Yang et al. found that adipose-specific* SLC2A4*-knockout mice show secondary insulin resistance in muscle and liver through elevated levels of retinol binding protein-4 (RBP4) in the serum. Further research revealed that increasing serum RBP4 induces hepatic expression of the gluconeogenic enzyme phosphoenolpyruvate carboxykinase 1 (PCK1) and reduces insulin-stimulated phosphoinositide 3-kinase (PI3K) activity in muscle. Thus, SLC2A4-RBP4-PCK1/PI3K is an adipocyte-derived signalling pathway that may contribute to the pathogenesis of T2DM [5].

Researchers have found that the levels of SLC2A4 in adipocytes of pregnant women with GDM were lower than those of normal pregnant women [6, 7]. Studies of RBP4 have suggested that the levels of RBP4 mRNA and serum RBP4 in adipocytes of pregnant women with GDM were higher than those of normal pregnant women with similar BMI [8–19]. Studies of PI3K have suggested that reducing PI3K levels can reduce the expression level of TRPM6 on the cell membrane and increase the risk of GDM [20]. These findings suggest that the SLC2A4-RBP4-PCK1/PI3K pathway not only is associated with T2DM, but also may be associated with the risk of GDM.

In this study, we investigated the association between GDM and* SLC2A4-RBP4-PCK1/PI3K *gene single nucleotide polymorphisms (SNPs) using a case-control research approach.

## 2. Subjects, Materials, and Methods

### 2.1. Ethics Statement

The study protocol was reviewed and approved by the Central-South University's Ethical and Confidentiality Committee. All participants provided written informed consent. The authors assert that all procedures/methods were conducted in accordance with the approved guidelines.

### 2.2. Study Population

This was a case–control study of pregnant women with and without GDM who enrolled on the oral glucose tolerance test (OGTT) day. The inclusion criteria for subjects were (a) visiting prenatal clinics regularly and undergoing OGTT during 24-28 weeks at the Department of Obstetrics and Gynecology in the Hunan Provincial Hospital of Maternal and Child Health from December 2014 to July 2015; (b) aged between 25 and 38 years; (c) singleton pregnancies; (d) without prepregnancy diabetes mellitus, hypertension, chronic liver disease, thyroid dysfunction or subclinical thyroid dysfunction, any known or suspected active infection, or other diseases, which are known risk factors for abnormal glucose metabolism; (e) no use of any medications except for minerals and vitamins. We diagnosed pregnant women with GDM according to the current GDM criteria in China. OGTT was done during 24-28 gestational weeks. The boundaries of OGTT were 5.1 mmol/L, 10.0 mmol/L, and 8.5 mmol/L for fasting glucose and 1 and 2 hours after 75 g oral glucose intake, respectively. When one or more OGTT indicators reached or exceeded the abovementioned boundaries, the pregnant woman was diagnosed with GDM. After obtaining informed consent, blood for genotyping of GDM pregnant women was obtained on OGTT afternoon when all the OGTT results came out. A similar number of women with normal glucose tolerance were randomly selected as the control group on the same afternoon and blood for genotyping was obtained. All subjects were collected for general information including maternal age, gestational age, parity, height, and weight (on OGTT morning and before pregnancy), and body mass index was calculated (BMI =body weight (kg)/body height (m) ^2^). Fasting insulin levels, systolic blood pressure, and diastolic blood pressure were measured on the OGTT morning. Gestational age was confirmed by a routine ultrasonographic examination performed during the first trimester of gestation.

### 2.3. SNP Selection and Genotyping

The candidate SNPs of* SLC2A4*,* RBP4*,* PCK1,* and* PIK3R1 *(coding gene of the PI3K P85 subunit) were selected by searching for the SNPs with the strongest signal in the literature (including GDM, T2DM, and metabolic syndrome) and selecting the tagSNP by searching the Genome Variation Server 141 (database search by gene name, population: HapMap-HCB, allele frequency cutoff (%)>10%, and R^2^ threshold for cluster: 0.8). Finally, 17 SNPs were selected. The alleles, minor allele frequency (MAF), and SNPs covered by tagSNP are shown in Table 1. The primers for each SNP are shown in Table S1.

Genomic DNA was extracted from whole blood using a TIANamp Blood DNA Kit (DP318-03, TIANGEN, Beijing), which is based on silica membrane technology and uses a special buffer system for DNA extraction from fresh or frozen whole blood. SNPs were genotyped with the SEQUENOM MassARRAY iPLEX platform. The assay consists of an initial locus-specific PCR reaction, followed by single-base extension and matrix-assisted laser desorption/ionization-time of flight mass spectrometry to identify the SNP allele.

### 2.4. Statistical Analysis

Case-control studies were conducted to compare the GDM and control groups. General clinical features of the case and control groups were compared with a t-test or the Mann-Whitney U test for continuous variables or the chi-square test for categorical variables. The Hardy-Weinberg test and linkage disequilibrium were estimated using SHEsis (http://analysis.bio-x.cn/myAnalysis.php)[21]. Pair-wise linkage disequilibrium parameters (D′ and r^2^) were estimated for* SLC2A4*,* RBP4*,* PCK1,* and* PIK3R1* genes. The most frequently used LD coefficients D′ and r^2^ have very different properties and may be applied for different purposes. D′ is useful to assess the probability for historical recombination in a given population, whereas r^2^ is useful in the context of association studies [22]. In this study, we mainly used r^2^ as the criterion for judging the linkage disequilibrium. When r^2^ was equal to or higher than 0.8, we judged that SNPs were in strong linkage disequilibrium. Meanwhile, if D′ was equal to 1, we judged that SNPs were in complete linkage disequilibrium. The chi-square test was used to compare the distribution of genotypes between the case and control groups. Logistic regression was used to estimate the odds ratio (OR) and 95% confidence interval (CI) of each SNP under different genetic models adjusted by maternal age, prepregnancy BMI, and weekly BMI growth. Linear regression was used to estimate the relationship between SNPs and the OGTT, fasting insulin, and homeostasis model assessment of insulin resistance (HOMA-IR) levels, adjusted by maternal age, prepregnancy BMI, and weekly BMI growth. The BMI measured on the morning of the OGTT minus the prepregnancy BMI and then divided by gestational age (weeks) was defined as “Weekly BMI growth.” HOMA-IR was calculated from the data of OGTT day. HOMA-IR=Fasting insulin (mIU/L)*∗* fasting blood glucose (mmol/L)/22.5.

All of the above statistical analyses, except for the Hardy-Weinberg test and linkage disequilibrium analysis, were performed using SPSS version 18.0 (SPSS Inc., Chicago, IL, USA). Model-based multifactor dimensionality reduction was used to estimate the adjusted interactions between genes [23, 24]. Even in the case of only one genotype combination distributed differently between the case and control groups, the specified genes had gene-gene interactions for GDM risk. The gene-gene interaction analysis was performed using R3.2.3, adjusting for maternal age, prepregnancy BMI, and weekly BMI growth. A multiple comparisons test with the Bonferroni correction was used to assess the significance level of the association. For the logistic regression and linear regression analysis, *α* was equal to 0.004 (0.004=0.05/14) because 14 SNPs were finally included in the analyses. Because three genetic models of each SNP have been analysed, a stringent Bonferroni correction was also applied, and *α* was equal to 0.001 (*α*=0.05/(14*∗*3)=0.001). *α* was set as 0.05.

## 3. Results

### 3.1. Demographic and Clinical Characteristics

A total of 334 cases and 367 controls were analysed. The clinical characteristics of cases and controls are summarized in Table 2. Compared with the control group, the case group had a higher prepregnancy BMI (*p*<0.001), larger weekly BMI growth (*p*<0.001), higher systolic blood pressure (*p*<0.001), higher diastolic blood pressure (*p*<0.001), and higher family history positive rate (*p*<0.001).

### 3.2. Test for Hardy-Weinberg Equilibrium and Linkage Disequilibrium Analysis

The SNP genotyping detection rate was 99.5%. For all SNPs, Hardy-Weinberg equilibrium (HWE) was observed in the control group (Table 1). Pair-wise linkage disequilibrium parameters (D′ and r^2^) were estimated for* SLC2A4*,* RBP4*,* PCK1,* and* PIK3R1* genes. For* SLC2A4*, rs222852 and rs5418 were in strong linkage disequilibrium. (Table S2). For* RBP4*, rs17108991, rs34571439, rs7079946, and rs7091052 were in complete linkage disequilibrium (Table S3). In the subsequent analysis, we included only rs7091052 and rs3758539. No pair of SNPs in* PCK1* and* PIK3R1 *genes was in strong linkage disequilibrium (Table S4, Table S5).

### 3.3. Association between Genetic Variants in SLC2A4, RBP4, PCK1, PIK3R1, and GDM

Table 3 shows that the frequencies of the* RBP4* rs7091052 T allele (*p*=0.012) and CT genotype (*p*=0.003) were significantly higher in the case than in the control group. After correction for multiple comparisons, the frequency of the rs7091052 CT genotype was still significantly higher in the case than in the control group (*α*=0.05/14=0.004). However, no significant differences in the alleles and genotypes of* SLC2A4*,* PCK1,* and* PIK3R1 *were observed between cases and controls (Table S6).

In the logistic regression analysis,* SLC2A4* rs5435, rs222852, rs5418, and rs8082645;* RBP4* rs3758539;* PCK1* rs1042531, rs2236745, rs28359554, and rs707555; and* PIK3R1* rs40419, rs1819987, rs34309, and rs6890176 were not associated with GDM, regardless of genotype and use of dominant model, recessive model, or additive model comparisons (Table S7).

As shown in Table 4, after adjusting for maternal age, prepregnancy BMI, and weekly BMI growth, the logistic regression analysis revealed that for cases, compared to controls,* RBP4* rs7091052 was significantly associated with GDM (Dominance model: OR=1.710,* p*=0.011, and 95%CI:[1.129,2.591]; Additive model: OR=1.493,* p*=0.041, and 95%CI: [1.017,2.191]).

### 3.4. Association Analysis of Genetic Variants in SLC2A4, RBP4, PCK1, and PIK3R1 with OGTT, Fasting Insulin, and HOMA-IR Levels

In addition to fasting blood glucose level, the blood glucose levels at 1 and 2 hours after the OGTT, which constitute the diagnostic criteria for GDM, fasting insulin, and HOMA-IR levels, are also important indicators for evaluating glucose metabolism. To better study the relationship between the selected genes and the glucose metabolism level, we analyzed the relationships between the fasting insulin level or other continuous indicators and genes. As shown in Table 5, after adjusting for maternal age, prepregnancy BMI, and weekly BMI growth, the linear regression analysis revealed that (1) the* SLC2A4* rs5434 TT genotype was associated with a higher fasting blood glucose level (Beta=0.171,* p*=0.042), fasting insulin level (Beta=3.166,* p*=0.002), and HOMA-IR level (Beta=0.879,* p*<0.001); (2) the* RBP4* rs7091052 TT and CT genotypes were associated with a higher 1-hour blood glucose level (Beta=0.542,* p*=0.030); (3) under the recessive model, the* PCK1* rs1042531 GG genotype was associated with higher fasting insulin (Beta=5.443,* p*<0.001) and HOMA-IR levels (Beta=1.485,* p*<0.001); under the additive model, the G mutation was still associated with higher fasting insulin (Beta=1.228,* p*=0.030) and HOMA-IR levels (Beta=0.341,* p*=0.014); (4) the* PCK1* rs2236745 CC and TC genotypes were associated with a higher fasting insulin level (Beta=1.571,* p*=0.032); (5) the* PIK3R1* rs34309 AA genotype was associated with higher fasting blood glucose (Beta=-0.222,* p*=0.011) and 1-hour blood glucose levels (Beta=-0.701,* p*=0.007). After stringent Bonferroni correction for multiple comparisons,* SLC2A4* rs5435 and* PCK1* rs1042531 were still associated with increased HOMA-IR and/or fasting insulin levels (*α*=0.05/(14*∗*3)=0.001). No significant results were observed for the association analysis of other SNPs with OGTT, fasting insulin, and HOMA-IR levels.

### 3.5. Gene-Gene Interaction in GDM

In the above analysis,* SLC2A4 *rs5435 (Recessive model);* RBP4* rs7091052 (Dominance model);* PCK1* rs1042531 (Recessive model) and rs2236745 (Dominance model); and* PIK3R1* rs34309 (Recessive model) were associated with GDM risk. We included* SLC2A4* rs5435,* RBP4* rs7091052,* PCK1* rs1042531 and rs2236745, and* PIK3R1* rs34309 in a gene-gene interaction analysis, adjusting for maternal age, prepregnancy BMI, and weekly BMI growth.

Compared to pregnant women with the other genotype combinations, pregnant women with* SLC2A4 *rs5435 (CC/CT),* RBP4 *rs7091052 (CC),* PCK1* rs1042531 (TT/TG) and rs2236745 (TT), and* PIK3R1 *rs34309 (AA) had a lower GDM risk (OR=0.231,* p*=0.012). The detailed data are shown in Table S8. Gene-gene interactions existed for* SLC2A4*,* RBP4*,* PCK1, *and* PI3K*.

## 4. Discussion

*SLC2A4 *rs5435;* RBP4 *rs7091052, rs17108991, rs34571439, and rs7079946;* PCK1 *rs1042531 and rs2236745; and* PIK3R1 *rs34309 were associated with GDM risk.

SLC2A4 is a glucose transporter that is the only insulin-sensitive protein in the glucose transporter family. When insulin binds to its receptor, the signal is passed down to SLC2A4, causing SLC2A4-rich vesicles to move towards the plasma membrane. As the vesicles fuse with the plasma membrane, SLC2A4 transporters are inserted and become available for transporting glucose, and glucose absorption increases [25]. In our study, we analysed the association of rs5435 with blood glucose and insulin and found that fasting blood glucose, fasting insulin, and HOMA-IR were higher in women with the TT genotype. The only previous correlation study showed that the T allele of rs5435 was associated with a high risk of T2DM [26].* SLC2A4* rs5435, a tagSNP, is located in the coding region, and the mutation is a synonymous mutation.* SLC2A4* rs5435 may affect glucose metabolism in pregnant women by influencing the level of mRNA and further modulating the protein level of SLC2A4, thus leading to a high risk of GDM [7].

RBP4 is mainly synthesized by hepatocytes and adipose tissue. It was identified in 2005 as an adipocytokine with the potential to reduce insulin sensitivity and enhance hepatic gluconeogenesis [5]. The results of our study showed that* RBP4* rs3758539 was not associated GDM risk. To date, three studies have focused on the relationship between* RBP4* and GDM risk, and all of three studies analysed rs3758539. In a study reported in the United States, rs3758539 was not associated with GDM risk [27]. A Mexican study also supported that rs3758539 was not associated with GDM risk; however, the A allele was associated with higher insulin and HOMA-IR levels six months after delivery [28]. Ping studied rs3758539 in a Chinese population and found that the A allele may reduce the risk of GDM [18]. However, the rs3758539 A allele was found to be associated with high insulin resistance levels in T2DM, metabolic syndrome, obesity, and lipid metabolism-related studies [29–33]. Therefore, more research is needed to confirm whether* RBP4* rs3758539 is associated with GDM risk and to determine the role of the A allele. Hu et al. found that the serum levels of RBP4 were higher in a Han population with* RBP4* rs7091052 TT and CT genotypes [34]. Our team performed a meta-analysis of the relationship between the risk of GDM and the serum RBP4 level, which showed that the serum levels of RBP4 in pregnant women with GDM were higher than those in normal pregnant women [35]. We inferred that the TT and CT genotypes of rs7091052 are high-risk genotypes for GDM, which was confirmed by the results of our study. The* RBP4* rs7091052 results, logistic regression analysis, and linear regression analysis suggested that RBP4 is likely part of the pathophysiology of GDM.

PCK1 is a gluconeogenic enzyme. In this study, we found that the GG genotype of rs1042531 was associated with higher levels of fasting insulin and HOMA-IR, suggesting that the GG genotype was a high-risk genotype of abnormal glucose metabolism; however, the GG genotype was not found to be associated with the risk of GDM in the single-factor logistic regression analysis. To date, the association between rs1042531 and glycometabolism has been explored only in T2DM patients, and the results are inconsistent. Studies of Chinese and South Asian-born British populations concluded that rs1042531 was not associated with T2DM risk [36, 37]. However, a study in the United States showed positive results, although the authors revealed that the G allele was a low-risk allele for T2DM risk [38]. Additional studies are needed to explore whether rs1042531 is associated with GDM risk and whether the influences are different among different races. This study found the CC and TC genotypes of rs2236745, a tagSNP, were related to higher fasting insulin levels. Although no relevant study has focused on this tagSNP, rs2071023, which is in high linkage disequilibrium with rs2236745, has been studied several times in T2DM patients in the UK, China, Finland, Canada, Japan, Denmark, and Germany [36, 37, 39–44]. In addition to studies that have reported that rs2071023 was not related to T2DM risk [37, 40, 43, 44], other studies have suggested that the minor allele was a high-risk allele for T2DM [36, 39, 41, 42], which was consistent with our study.

PI3K is a key effector of the insulin signalling pathway that can affect the movement of SLC2A4 in skeletal muscle and inhibit liver gluconeogenesis. PI3K is composed of a P85 subunit and a P110 subunit. P85 and the P85-P110 complex compete for phosphotyrosine sites of insulin receptor substrate-1, while P85 activates phosphatase and tonic protein homologues, attenuating the insulin signal. The balance between P85 and P110 is critical for the insulin signalling PI3K pathway [45–47]. In this study, we focused on the* PIK3R1 *gene, which is the coding gene of the PI3K P85 subunit. Thus far, only one study has focused on* PIK3R1* and GDM risk (Italy), suggesting that rs3729982 is not associated with the risk of GDM [48]. However, the sample size of the Italian study was relatively small (240 controls, 38 pregnant women with GDM). The four SNPs included in this study were all tagSNP. The results showed that the GG and GA genotypes of rs34309 were related to higher levels of fasting blood glucose and blood glucose at the 1-hour OGTT, suggesting that the P85 subunit of PI3K may be related to abnormal glucose metabolism. We suggested that SNPs of the PI3K P85 subunit are associated with the risk of GDM, which need to be tested in larger samples.

This study revealed that gene-gene interactions related to GDM risk existed for* SLC2A4*,* RBP4*,* PCK1,* and* PI3K*. This finding suggested that these four proteins may be part of a pathway that affects GDM risk. The potential mechanism of the interaction may be that RBP4 protein levels can be regulated by SLC2A4 protein levels. Simultaneously, RBP4 protein level can regulate the protein levels of PCK1 and PI3K. Protein levels can be regulated by the encoding gene and exerts a feedback regulation function on the transcription and translation of the encoding gene. However, due to the relatively small sample size, the analysis of interactions in this paper is a preliminary study. The results were not conclusive but may be indicative. We will recruit additional patients in the future to perform a study with sufficient power to verify the effects of gene-gene interactions on GDM.

The study has certain limitations. First, the genetic susceptibility analysis provided limited information about the association with GDM; the results need to be validated at other levels, such as the proteomics level. Second, because the study population was one race and the sample size was relatively small, the results need to be confirmed in other races and larger samples.

## 5. Conclusions

Our study showed that T2DM-related SNPs were associated with GDM in a Han Chinese population. The* SLC2A4*,* RBP4*,* PCK1, *and* PIK3R1* genes may be involved in common elements of the pathogenesis of T2DM and GDM. These results also provide genetic evidence to support that patients with GDM might have a higher risk for T2DM.

## Figures and Tables

**Table 1:** Information for selected SNPs.

Gene	dbSNP ID	Functional Consequence	Alleles^*∗∗*^	HWE p value	MAF	SNPs covered by tagSNP
*SLC2A4*	rs222852^*∗*^	intron variant	GA	0.955	0.38	
	rs5418^#^	UTR variant 5 prime	GA	0.935	0.40	rs5417, rs2654185
	rs5435^#^	missense,synonymous codon	CT	0.492	0.33	rs222849
	rs8082645^*∗*^	UTR variant 3 prime	TG	0.882	0.41	
*RBP4*	rs17108991^#^	intron variant	TG	0.091	0.11	rs13376835
	rs34571439^*∗*^	downstream variant 500B	TG	0.251	0.12	
	rs3758539^*∗*^	upstream variant 2KB	GA	0.358	0.13	
	rs7079946^#^	intron variant	TA	0.194	0.15	rs7094671
	rs7091052^*∗*^	intron variant	CT	0.078	0.14	
*PCK1*	rs1042531^*∗*^	UTR variant 3 prime	TG	0.113	0.19	
	rs2236745^#^	intron variant	TC	0.875	0.43	rs1328756, rs1328757, rs2071023, rs2236744
	rs28359554^*∗*^	UTR variant 3 prime	TC	0.494	0.29	
	rs707555^#^	missense	CG	0.623	0.22	rs2070756
*PIK3R1*	rs1819987^#^	intron variant	GC	0.993	0.41	rs2161120, rs2302975, rs6861401, rs6876003, rs6890202, rs10940160
	rs34309^#^	intron variant	GA	0.213	0.32	rs171649, rs173703, rs251408, rs831229, rs863818, rs2431166, rs2431167
	rs40419^#^	intron variant	CT	0.639	0.19	rs251398, rs706711, rs831227, rs10940157, rs13173003
	rs6890176^#^	intron variant	GA	0.806	0.18	rs2302976, rs6863431,rs6893676, rs10515074, rs10940159, rs12656176, rs16897558, rs16897601

^*∗*^These SNPs were found to be associated with GDM, T2DM, or metabolic syndrome risk in previous studies. #These SNPs were tagSNPs. ^*∗∗*^The second allele was the minor allele.

**Table 2:** Demographic and clinical characteristics of the study subjects.

	Controls (N=367)	Cases (N=334)	*p*
Maternal age, years	29(28,32)	29(27,32)	0.672^*∗*^
Gestational age at sampling, weeks	25.11±2.724	25.35±2.948	0.458^*∗∗*^
Pre-pregnancy BMI	20.55(19.14,22.64)	22.31(20.29,24.14)	**<0.001** ^*∗*^
Weekly BMI growth^a^	0.114±0.054	0.131±0.056	**<0.001** ^*∗∗*^
SBP^b^	111±10.30	116±11.12	**<0.001** ^*∗∗*^
DBP^b^	70±8.38	74±8.09	**<0.001** ^*∗∗*^
Parity			
0	230(62.7%)	216(64.7%)	0.312^*∗∗∗*^
1	123(33.5%)	93(27.8%)	
2	5(1.4%)	7(2.1%)	
Family history of diabetes^c^			
Yes	62(17.4%)	94(29.3%)	**<0.001** ^*∗∗∗*^
No	295(82.6%)	227(70.7%)	

^*∗*^ The Wilcoxon rank sum test was used due to a nonnormal distribution of the tested characteristics, and data are presented as medians and quartiles. ^*∗∗*^ Student's *t*-test was used due to a normal distribution of the tested characteristics, and data are presented as the mean and SDs. ^*∗∗∗*^ A Chi-square test was used to analyse data presented as a ratio. ^a^ BMI measured on the morning of the oral glucose tolerance test minus the prepregnancy BMI and then divided by the gestational age (weeks) was defined as “Weekly BMI growth”; ^b^ SBP (systolic blood pressure) and DBP (diastolic blood pressure) were the blood pressures measured on the morning of the oral glucose tolerance test. ^c^Relatives covered grandfather, grandmother, maternal grandfather, maternal grandmother, father, mother, brother, sister, and brother and sister of father and mother.

**Table 3:** The distribution of alleles and genotypes of *RBP4* rs7091052.

Gene	SNP	Allele/Genotype	Controls		Cases		*χ* ^2^	*p*
			n	%	n	%		
*RBP4*	rs7091052	C	672	91.8	586	87.7	**6.375**	**0.012**
		T	60	8.2	82	12.3		
		CC	311	85.0	254	76.0	**11.723**	**0.003** ^**∗**^
		TT	5	1.4	2	0.6		
		CT	50	13.7	78	23.4		

^*∗*^ The *p* value was less than or equal to 0.004, which was *α* after Bonferroni correction (*α*=0.05/14=0.004). Fourteen SNPs were included in the analyses.

**Table 4:** Logistic regression analysis of *RBP4* rs7091052 and GDM risk.

Gene	SNP	Genotype	OR	*p*	95%CI
*RBP4*	rs7091052	Recessive model	0.314	0.211	0.051,1.933
		Dominance model	1.710	**0.011**	1.129,2.591
		Additive model	1.493	**0.041**	1.017,2.191

The covariates in the logistic regression analysis were maternal age, prepregnancy BMI, and weekly BMI growth.

**Table 5:** The association of genetic variants of *SLC2A4*, *RBP4*, *PCK1,* and *PIK3R1* with OGTT, fasting insulin, and HOMA-IR levels.

Gene	SNP	Genetic model	Fasting BG^#^		1 h BG^#^		2 h BG^#^		Fasting insulin		HOMA-IR	
			Beta	*p*	Beta	*p*	Beta	*p*	Beta	*p*	Beta	*p*
*SLC2A4*	rs5435	Recessive model	0.171	**0.042**	0.338	0.180	0.287	0.169	3.166	**0.002** ^*∗*^	0.879	**<0.001** ^*∗*§^
*RBP4*	rs7091052	Dominance model	0.109	0.195	0.542	**0.030**	0.004	0.987	-0.060	0.927	0.025	0.890
*PCK1*	rs1042531	Recessive model	0.176	0.133	0.455	0.185	0.428	0.134	5.443	**<0.001** ^*∗*§^	1.485	**<0.001** ^*∗*§^
		Additive model	0.052	0.271	0.089	0.520	0.139	0.230	1.228	**0.030**	0.341	**0.014**
*PCK1*	rs2236745	Dominance model	0.040	0.515	-0.056	0.755	-0.002	0.989	1.571	**0.032**	0.352	0.051
*PIK3R1*	rs34309	Recessive model	-0.222	**0.011**	-0.701	**0.007**	-0.395	0.069	0.250	0.813	-0.088	0.735

The covariates in these linear regression analyses were maternal age, prepregnancy BMI, and weekly BMI growth. BG^#^ is the abbreviation for blood glucose. ^*∗*^*p* value was less than 0.004, which was *α* after Bonferroni correction (*α*=0.05/14=0.004); fourteen SNPs were included in the analyses. § meant that the *p* value was less than 0.001, which was *α* after stringent Bonferroni correction (*α*=0.05/(14*∗*3)=0.001); three genetic models of each SNP have been analysed.

## Data Availability

The data used to support the findings of this study are available from the corresponding author upon request.

## Supplementary Materials

Supplementary Materials Table S1. Primers of the selected SNPs. Table S2. Pair-wise linkage disequilibrium analyses of* SLC2A4* rs222852, rs5418, rs5435, and rs8082645. Table S3. Pair-wise linkage disequilibrium analyses of* RBP4* rs17108991, rs34571439, rs3758539, rs7079946, and rs7091052. Table S4. Pair-wise linkage disequilibrium analyses of* PCK1* rs1042531, rs2236745, rs28359554, and rs707555. Table S5. Pair-wise linkage disequilibrium analyses of* PIK3R1* rs1819987, rs34309, rs40419, and rs6890176. Table S6. The distribution of alleles and genotypes of* RBP4* rs7091052. Table S7. Logistic regression analyses of* SLC2A4* rs222852, rs5418, rs5435, and rs8082645;* RBP4* rs3758539;* PCK1* rs1042531, rs2236745, rs28359554, and rs707555;* PIK3R1* rs40419, rs1819987, rs34309, and rs6890176 with GDM risk. Table S8. Gene-gene interaction in GDM.
