# Supplementary material for: Relationships of SLC2A4, RBP4, PCK1, and PI3K Gene Polymorphisms with Gestational Diabetes Mellitus in a Chinese Population
Source: Biomed Res Int. 2019 Jan 20;2019:7398063. doi: 10.1155/2019/7398063 (PMC6363241; doi:10.1155/2019/7398063)
Supplement: Supplementary Materials — Table S1. Primers of the selected SNPs. Table S2. Pair-wise linkage disequilibrium analyses of SLC2A4 rs222852, rs5418, rs5435, and rs8082645. Table S3. Pair-wise linkage disequilibrium analyses of RBP4 rs17108991, rs34571439, rs3758539, rs7079946, and rs7091052. Table S4. Pair-wise linkage disequilibrium analyses of PCK1 rs1042531, rs2236745, rs28359554, and rs707555. Table S5. Pair-wise linkage disequilibrium analyses of PIK3R1 rs1819987, rs34309, rs40419, and rs6890176. Table S6. The distribution of alleles and genotypes of RBP4 rs7091052. Table S7. Logistic regression analyses of SLC2A4 rs222852, rs5418, rs5435, and rs8082645; RBP4 rs3758539; PCK1 rs1042531, rs2236745, rs28359554, and rs707555; PIK3R1 rs40419, rs1819987, rs34309, and rs6890176 with GDM risk. Table S8. Gene-gene interaction in GDM. [file 7398063.f1.doc]

**Table S1. Primers of the selected SNPs.**

| Gene | SNP | 5'utr region primers | 3'utr region primers |
| --- | --- | --- | --- |
| *SLC2A4* | rs222852 | ACGTTGGATGTTTCCTACCACAGCCCTAAG | ACGTTGGATGTGGTGGGACTATAGAGTGAC |
|  | rs5418 | ACGTTGGATGATGGGACCCACAGCCACAAG | ACGTTGGATGGGCTTCTCGCGTCTTTTCC |
|  | rs5435 | ACGTTGGATGCGTCCAAGGATGAGCATTTC | ACGTTGGATGCATGCTGGTCAACAATGTCC |
|  | rs8082645 | ACGTTGGATGAATTCTTGCCACGCAGACTC | ACGTTGGATGGGAGACAGCTTGCAGTGAAG |
| *RBP4* | rs17108991 | ACGTTGGATGTGAGATTCTCTGGAACAACC | ACGTTGGATGGAGTGAGTGTCCACTCAAAC |
|  | rs34571439 | ACGTTGGATGTCAAATCCAGCACTGGTGAG | ACGTTGGATGGAAGTGAGTAGACAGGTCAG |
|  | rs3758539 | ACGTTGGATGTCTCTTTCAGGAGCGTTGTG | ACGTTGGATGGGTTGCGTTTCTGGAGAATA |
|  | rs7079946 | ACGTTGGATGTGAGTTGAGGTTGCACCACT | ACGTTGGATGTTGGTGTAGTGGGTCAGTTC |
|  | rs7091052 | ACGTTGGATGCCTCTGCAGTGTTCCAGTTG | ACGTTGGATGGCAACTTAGGACCAGCATTC |
| *PCK1* | rs1042531 | ACGTTGGATGTGTTCCCAAATTGACGCCAC | ACGTTGGATGTTAGCTACTACCCAGTGTTC |
|  | rs2236745 | ACGTTGGATGAAAAAAAGGCAGCCCCTCTC | ACGTTGGATGTGAGCACGCTAACAACAGTC |
|  | rs28359554 | ACGTTGGATGACTAGTAGGTCATCTTGCCC | ACGTTGGATGTGCACATGTGTCTGTGTGGT |
|  | rs707555 | ACGTTGGATGAAGATCGGCATCGAGCTGAC | ACGTTGGATGAGGCATTTGACAAACTCCCC |
| *PIK3R1* | rs1819987 | ACGTTGGATGTAGAAGCACAAACGACTGGC | ACGTTGGATGATTTCTAGCACGTCCTCCAG |
|  | rs34309 | ACGTTGGATGAAACAGCTTTGACTCAGCAG | ACGTTGGATGGTGAAAAACTGCCACTTACC |
|  | rs40419 | ACGTTGGATGTGTTTGCAGAGTTAAGAGGC | ACGTTGGATGATTGAATGTCACCTACTGCC |
|  | rs6890176 | ACGTTGGATGAAAGTCTCAGAAGTTGCCTC | ACGTTGGATGTCAGTTTTGGACTCCGTCAG |

**Table S2.** Pair-wise linkage disequilibrium analyses of *SLC2A4* rs222852, rs5418, rs5435 and rs8082645.

| Group | SNP | rs5418 | |  | rs5435 | |  | rs8082645 | |
| --- | --- | --- | --- | --- | --- | --- | --- | --- | --- |
|  |  | D' | r^2^ |  | D' | r^2^ |  | D' | r^2^ |
| Controls | rs222852 | 0.955 | 0.838 |  | 0.745 | 0.166 |  | 0.908 | 0.691 |
|  | rs5418 | - | - |  | 0.863 | 0.205 |  | 0.906 | 0.747 |
|  | rs5435 | - | - |  | - | - |  | 0.982 | 0.245 |
| Cases | rs222852 | 0.956 | 0.842 |  | 0.888 | 0.259 |  | 0.918 | 0.771 |
|  | rs5418 | - | - |  | 0.936 | 0.269 |  | 0.917 | 0.835 |
|  | rs5435 | - | - |  | - | - |  | 1.000 | 0.305 |

**Table S3.** Pair-wise linkage disequilibrium analyses of *RBP4* rs17108991, rs34571439, rs3758539, rs7079946 and rs7091052.

| Group | SNP | rs34571439 | |  | rs3758539 | |  | rs7079946 | |  | rs7091052 | |
| --- | --- | --- | --- | --- | --- | --- | --- | --- | --- | --- | --- | --- |
|  |  | D' | r^2^ |  | D' | r^2^ |  | D' | r^2^ |  | D' | r^2^ |
| Controls | rs17108991 | 1.000 | 0.984 |  | 0.964 | 0.854 |  | 1.000 | 0.896 |  | 1.000 | 0.886 |
|  | rs34571439 | - | - |  | 0.964 | 0.854 |  | 1.000 | 0.896 |  | 1.000 | 0.886 |
|  | rs3758539 | - | - |  | - | - |  | 0.963 | 0.764 |  | 0.871 | 0.732 |
|  | rs7079946 | - | - |  | - | - |  | - | - |  | 1.000 | 0.794 |
| Cases | rs17108991 | 1.000 | 1.000 |  | 0.968 | 0.777 |  | 1.000 | 0.937 |  | 1.000 | 0.934 |
|  | rs34571439 | - | - |  | 0.968 | 0.780 |  | 1.000 | 0.937 |  | 1.000 | 0.934 |
|  | rs3758539 | - | - |  | - | - |  | 0.968 | 0.730 |  | 0.921 | 0.756 |
|  | rs7079946 | - | - |  | - | - |  | - | - |  | 1.000 | 0.876 |

**Table S4.** Pair-wise linkage disequilibrium analyses of *PCK1* rs1042531, rs2236745, rs28359554 and rs707555.

| Group | SNP | rs2236745 | |  | rs28359554 | |  | rs707555 | |
| --- | --- | --- | --- | --- | --- | --- | --- | --- | --- |
|  |  | D' | r^2^ |  | D' | r^2^ |  | D' | r^2^ |
| Controls | rs1042531 | 0.351 | 0.068 |  | 0.848 | 0.080 |  | 0.896 | 0.076 |
|  | rs2236745 | - | - |  | 0.850 | 0.146 |  | 0.973 | 0.163 |
|  | rs28359554 | - | - |  | - | - |  | 0.999 | 0.085 |
| Cases | rs1042531 | 0.233 | 0.030 |  | 0.703 | 0.056 |  | 0.884 | 0.082 |
|  | rs2236745 | - | - |  | 0.866 | 0.153 |  | 1.000 | 0.193 |
|  | rs28359554 | - | - |  | - | - |  | 0.875 | 0.058 |

**Table S5.** Pair-wise linkage disequilibrium analyses of *PIK3R1* rs1819987, rs34309, rs40419 and rs6890176.

| Group | SNP | rs34309 | |  | rs40419 | |  | rs6890176 | |
| --- | --- | --- | --- | --- | --- | --- | --- | --- | --- |
|  |  | D' | r^2^ |  | D' | r^2^ |  | D' | r^2^ |
| Controls | rs1819987 | 1.000 | 0.398 |  | 0.125 | 0.004 |  | 1.000 | 0.377 |
|  | rs34309 | - | - |  | 0.406 | 0.109 |  | 1.000 | 0.150 |
|  | rs40419 | - | - |  | - | - |  | 0.544 | 0.029 |
| Cases | rs1819987 | 0.986 | 0.363 |  | 0.085 | 0.002 |  | 1.000 | 0.339 |
|  | rs34309 | - | - |  | 0.245 | 0.039 |  | 1.000 | 0.126 |
|  | rs40419 | - | - |  | - | - |  | 0.771 | 0.050 |

**Table S6.** The distribution of alleles and genotypes.

| Gene | SNP | Allele | Controls | |  | Cases | | | |
| --- | --- | --- | --- | --- | --- | --- | --- | --- | --- |
|  |  |  | n | % |  | n | % | χ^2^ | *p* |
| *SLC2A4* | rs222852 | G | 469 | 64.1 |  | 434 | 66.0 | 0.542 | 0.462 |
|  |  | A | 263 | 35.9 |  | 224 | 34.0 |  |  |
|  |  | GG | 150 | 41.0 |  | 142 | 43.2 | 0.573 | 0.751 |
|  |  | AA | 47 | 12.8 |  | 37 | 11.2 |  |  |
|  |  | GA | 169 | 46.2 |  | 150 | 45.6 |  |  |
|  | rs5418 | G | 483 | 66.0 |  | 448 | 67.1 | 0.184 | 0.668 |
|  |  | A | 249 | 34.0 |  | 220 | 32.9 |  |  |
|  |  | GG | 159 | 43.4 |  | 155 | 46.4 | 1.008 | 0.604 |
|  |  | AA | 42 | 11.5 |  | 41 | 12.3 |  |  |
|  |  | GA | 165 | 45.1 |  | 138 | 41.3 |  |  |
|  | rs5435 | C | 477 | 65.0 |  | 411 | 61.5 | 1.802 | 0.179 |
|  |  | T | 257 | 35.9 |  | 257 | 38.5 |  |  |
|  |  | CC | 152 | 41.4 |  | 131 | 39.2 | 3.301 | 0.192 |
|  |  | TT | 42 | 11.4 |  | 54 | 16.2 |  |  |
|  |  | CT | 173 | 47.1 |  | 149 | 44.6 |  |  |
|  | rs8082645 | T | 499 | 68.0 |  | 449 | 67.2 | 0.094 | 0.759 |
|  |  | G | 235 | 32.0 |  | 219 | 32.8 |  |  |
|  |  | TT | 169 | 46.0 |  | 157 | 47.0 | 1.492 | 0.474 |
|  |  | GG | 37 | 10.1 |  | 42 | 12.6 |  |  |
|  |  | TG | 161 | 43.9 |  | 135 | 40.4 |  |  |
| *RBP4* | rs3758539 | G | 670 | 91.5 |  | 594 | 88.9 | 2.708 | 0.100 |
|  |  | A | 62 | 8.5 |  | 74 | 11.1 |  |  |
|  |  | GG | 308 | 84.2 |  | 262 | 78.4 | 4.991 | 0.083 |
|  |  | AA | 4 | 1.1 |  | 2 | 0.6 |  |  |
|  |  | GA | 54 | 14.8 |  | 70 | 21.0 |  |  |
| *PCK1* | rs1042531 | T | 543 | 74.0 |  | 479 | 71.7 | 0.914 | 0.339 |
|  |  | G | 191 | 26.0 |  | 189 | 28.3 |  |  |
|  |  | TT | 195 | 53.1 |  | 171 | 51.2 | 1.996 | 0.369 |
|  |  | GG | 19 | 5.2 |  | 26 | 7.8 |  |  |
|  |  | TG | 153 | 41.7 |  | 137 | 41.0 |  |  |
|  | rs2236745 | T | 448 | 61.0 |  | 385 | 58.0 | 1.350 | 0.245 |
|  |  | C | 286 | 39.0 |  | 279 | 42.0 |  |  |
|  |  | TT | 136 | 37.1 |  | 108 | 32.5 | 1.607 | 0.448 |
|  |  | CC | 55 | 15.0 |  | 55 | 16.6 |  |  |
|  |  | TC | 176 | 48.0 |  | 169 | 50.9 |  |  |
|  | rs28359554 | T | 555 | 75.8 |  | 520 | 77.8 | 0.803 | 0.370 |
|  |  | C | 177 | 24.2 |  | 148 | 22.2 |  |  |
|  |  | TT | 208 | 56.8 |  | 202 | 60.5 | 0.959 | 0.619 |
|  |  | CC | 19 | 5.2 |  | 16 | 4.8 |  |  |
|  |  | TC | 139 | 38.0 |  | 116 | 34.7 |  |  |
|  | rs707555 | C | 578 | 78.7 |  | 527 | 78.9 | 0.004 | 0.947 |
|  |  | G | 156 | 21.3 |  | 141 | 21.1 |  |  |
|  |  | CC | 226 | 61.6 |  | 204 | 61.1 | 0.388 | 0.824 |
|  |  | GG | 15 | 4.1 |  | 11 | 3.3 |  |  |
|  |  | CG | 126 | 34.3 |  | 119 | 35.6 |  |  |
| *PIK3R1* | rs1819987 | G | 426 | 58.2 |  | 382 | 57.5 | 0.063 | 0.801 |
|  |  | C | 306 | 41.8 |  | 282 | 42.5 |  |  |
|  |  | GG | 124 | 33.9 |  | 115 | 34.6 | 0.741 | 0.690 |
|  |  | CC | 64 | 17.5 |  | 65 | 19.6 |  |  |
|  |  | GC | 178 | 48.6 |  | 152 | 45.8 |  |  |
|  | rs34309 | G | 471 | 64.3 |  | 443 | 66.7 | 0.867 | 0.352 |
|  |  | A | 261 | 35.7 |  | 221 | 33.3 |  |  |
|  |  | GG | 157 | 42.9 |  | 146 | 44.0 | 2.187 | 0.335 |
|  |  | AA | 52 | 14.2 |  | 35 | 10.5 |  |  |
|  |  | GA | 157 | 42.9 |  | 151 | 45.5 |  |  |
|  | rs40419 | C | 536 | 73.2 |  | 501 | 75.0 | 0.574 | 0.449 |
|  |  | T | 196 | 26.8 |  | 167 | 25.0 |  |  |
|  |  | CC | 198 | 54.1 |  | 189 | 56.6 | 0.566 | 0.753 |
|  |  | TT | 28 | 7.7 |  | 22 | 6.6 |  |  |
|  |  | CT | 140 | 38.3 |  | 123 | 36.8 |  |  |
|  | rs6890176 | G | 577 | 78.6 |  | 533 | 80.0 | 0.429 | 0.513 |
|  |  | A | 157 | 21.4 |  | 133 | 20.0 |  |  |
|  |  | GG | 226 | 61.6 |  | 212 | 63.7 | 0.463 | 0.794 |
|  |  | AA | 16 | 4.4 |  | 12 | 3.6 |  |  |
|  |  | GA | 125 | 34.1 |  | 109 | 32.7 |  |  |

**Table S7.** Logistic regression analyses of *SLC2A4* rs222852, rs5418, rs5435 and rs8082645; *RBP4* rs3758539*; PCK1* rs1042531, rs2236745, rs28359554, and rs707555, *PIK3R1* rs40419, rs1819987, rs34309 and rs6890176 with GDM risk.

| Gene | SNP | Genotype | OR | *p* | 95%CI |
| --- | --- | --- | --- | --- | --- |
| *SLC2A4* | rs222852 | Recessive model | 0.907 | 0.697 | 0.554,1.485 |
|  |  | Dominance model | 0.981 | 0.910 | 0.709,1.358 |
|  |  | Additive model | 0.968 | 0.786 | 0.763,1.228 |
|  | rs5418 | Recessive model | 1.119 | 0.657 | 0.682,1.834 |
|  |  | Dominance model | 0.961 | 0.808 | 0.697,1.325 |
|  |  | Additive model | 1.004 | 0.973 | 0.793,1.271 |
|  | rs5435 | Recessive model | 1.317 | 0.243 | 0.829,2.093 |
|  |  | Dominance model | 1.037 | 0.825 | 0.749,1.436 |
|  |  | Additive model | 1.092 | 0.457 | 0.866,1.377 |
|  | rs8082645 | Recessive model | 1.481 | 0.132 | 0.889,2.469 |
|  |  | Dominance model | 1.024 | 0.886 | 0.743,1.410 |
|  |  | Additive model | 1.104 | 0.416 | 0.870,1.401 |
| *RBP4* | rs3758539 | Recessive model | 0.426 | 0.384 | 0.062,2.913 |
|  |  | Dominance model | 1.432 | 0.091 | 0.944,2.172 |
|  |  | Additive model | 1.317 | 0.165 | 0.893,1.943 |
| *PCK1* | rs1042531 | Recessive model | 1.255 | 0.492 | 0.656,2.400 |
|  |  | Dominance model | 0.973 | 0.868 | 0.706,1.341 |
|  |  | Additive model | 1.019 | 0.886 | 0.786,1.322 |
|  | rs2236745 | Recessive model | 1.166 | 0.493 | 0.752,1.180 |
|  |  | Dominance model | 1.261 | 0.180 | 0.899,1.769 |
|  |  | Additive model | 1.169 | 0.194 | 0.924,1.479 |
|  | rs28359554 | Recessive model | 0.855 | 0.684 | 0.404,1.813 |
|  |  | Dominance model | 0.829 | 0.262 | 0.598,1.150 |
|  |  | Additive model | 0.859 | 0.278 | 0.654,1.130 |
|  | rs707555 | Recessive model | 0.805 | 0.617 | 0.344,1.885 |
|  |  | Dominance model | 1.110 | 0.534 | 0.799,1.542 |
|  |  | Additive model | 1.055 | 0.713 | 0.794,1.400 |
| *PIK3R1* | rs1819987 | Recessive model | 1.128 | 0.572 | 0.743,1.714 |
|  |  | Dominance model | 0.974 | 0.876 | 0.695,1.363 |
|  |  | Additive model | 1.024 | 0.840 | 0.815,1.285 |
|  | rs34309 | Recessive model | 0.639 | 0.068 | 0.383,1.035 |
|  |  | Dominance model | 1.012 | 0.945 | 0.732,1.397 |
|  |  | Additive model | 0.904 | 0.402 | 0.714,1.145 |
|  | rs40419 | Recessive model | 0.702 | 0.276 | 0.371,1.326 |
|  |  | Dominance model | 0.922 | 0.620 | 0.668,1.272 |
|  |  | Additive model | 0.896 | 0.401 | 0.693,1.158 |
|  | rs6890176 | Recessive model | 0.905 | 0.811 | 0.400,2.047 |
|  |  | Dominance model | 0.889 | 0.488 | 0.639,1.239 |
|  |  | Additive model | 0.908 | 0.501 | 0.685,1.204 |

**Table S8.** Gene-gene interaction in GDM.

| rs34309 | rs2236745 | rs1042531 | rs7091052 | rs5435 | Cases | Controls | OR | *p* |
| --- | --- | --- | --- | --- | --- | --- | --- | --- |
| GG/GA | CC/TC | GG | TT/CT | TT | 3 | 0 | 1513583.276 | 0.976 |
| GG/GA | TT | GG | TT/CT | TT | 1 | 0 | 165213.180 | 0.982 |
| AA | CC/TC | TT/TG | TT/CT | TT | 1 | 1 | 0.000 | 0.979 |
| GG/GA | CC/TC | TT/TG | TT/CT | TT | 7 | 4 | 1.881 | 0.340 |
| AA | TT | TT/TG | TT/CT | TT | 1 | 0 | 1.000 | 1.000 |
| GG/GA | TT | TT/TG | TT/CT | TT | 2 | 2 | 0.893 | 0.910 |
| GG/GA | CC/TC | GG | CC | TT | 4 | 2 | 1.685 | 0.561 |
| AA | TT | GG | CC | TT | 0 | 1 | 0.000 | 0.979 |
| GG/GA | TT | GG | CC | TT | 1 | 0 | 1188248.095 | 0.979 |
| AA | CC/TC | TT/TG | CC | TT | 0 | 1 | 0.000 | 0.982 |
| GG/GA | CC/TC | TT/TG | CC | TT | 21 | 18 | 1.172 | 0.651 |
| AA | TT | TT/TG | CC | TT | 1 | 0 | 602589.239 | 0.980 |
| GG/GA | TT | TT/TG | CC | TT | 12 | 12 | 1.075 | 0.870 |
| GG/GA | CC/TC | GG | TT/CT | CC/CT | 4 | 0 | 2566336.807 | 0.972 |
| AA | CC/TC | TT/TG | TT/CT | CC/CT | 5 | 5 | 1.026 | 0.971 |
| GG/GA | CC/TC | TT/TG | TT/CT | CC/CT | 35 | 28 | 1.522 | 0.150 |
| AA | TT | TT/TG | TT/CT | CC/CT | 1 | 3 | 0.221 | 0.266 |
| GG/GA | TT | TT/TG | TT/CT | CC/CT | 20 | 12 | 1.677 | 0.201 |
| AA | CC/TC | GG | CC | CC/CT | 1 | 3 | 0.317 | 0.322 |
| GG/GA | CC/TC | GG | CC | CC/CT | 10 | 10 | 0.895 | 0.821 |
| GG/GA | TT | GG | CC | CC/CT | 2 | 2 | 1.050 | 0.962 |
| AA | CC/TC | TT/TG | CC | CC/CT | 21 | 20 | 1.170 | 0.650 |
| GG/GA | CC/TC | TT/TG | CC | CC/CT | 111 | 139 | 0.923 | 0.642 |
| **AA** | **TT** | **TT/TG** | **CC** | **CC/CT** | **4** | **18** | **0.231** | **0.012** |
| GG/GA | TT | TT/TG | CC | CC/CT | 62 | 85 | 0.791 | 0.253 |
